# Is *RNASEL*:p.Glu265* a modifier of early-onset breast cancer risk for carriers of high-risk mutations?

**DOI:** 10.1186/s12885-018-4028-z

**Published:** 2018-02-08

**Authors:** Tú Nguyen-Dumont, Zhi L. Teo, Fleur Hammet, Alexis Roberge, Maryam Mahmoodi, Helen Tsimiklis, Daniel J. Park, Bernard J. Pope, Andrew Lonie, Miroslav K. Kapuscinski, Khalid Mahmood, ABCFR, David E. Goldgar, Graham G. Giles, Ingrid Winship, John L. Hopper, Melissa C. Southey

**Affiliations:** 1 Genetic Epidemiology Laboratory, Department of Clinical Pathology, The University of Melbourne, Melbourne, VIC Australia; 2 Precision Medicine, School of Clinical Sciences at Monash Health, Monash University, Clayton, VIC Australia; 3 Peter MacCallum Cancer Centre, Melbourne, VIC Australia; 4 Sir Peter MacCallum Department of Oncology, The University of Melbourne, Melbourne, VIC Australia; 5 Melbourne Bioinformatics, The University of Melbourne, Melbourne, VIC Australia; 6 Department of Clinical Pathology, The University of Melbourne, Melbourne, VIC Australia; 7 Department of Medicine, School of Clinical Sciences at Monash Health, Monash University, Clayton, VIC Australia; 8 Centre for Epidemiology and Biostatistics, Melbourne School of Population and Global Health, The University of Melbourne, Melbourne, VIC Australia; 9 Huntsman Cancer Institute, Salt Lake City, UT USA; 10 Cancer Epidemiology & Intelligence Division, Cancer Council Victoria, Melbourne, VIC Australia; 11 Department of Medicine, The University of Melbourne, Melbourne, VIC Australia; 12 The Royal Melbourne Hospital, Melbourne, VIC Australia

**Keywords:** *RNASEL:*P.Glu265*, Breast cancer, Modifier risk gene, Early-onset cancer

## Abstract

**Background:**

Breast cancer risk for *BRCA1* and *BRCA2* pathogenic mutation carriers is modified by risk factors that cluster in families, including genetic modifiers of risk. We considered genetic modifiers of risk for carriers of high-risk mutations in other breast cancer susceptibility genes.

**Methods:**

In a family known to carry the high-risk mutation *PALB2*:c.3113G>A (p.Trp1038*), whole-exome sequencing was performed on germline DNA from four affected women, three of whom were mutation carriers.

**Results:**

*RNASEL:*p.Glu265* was identified in one of the *PALB2* carriers who had two primary invasive breast cancer diagnoses before 50 years. Gene-panel testing of *BRCA1*, *BRCA2, PALB2* and *RNASEL* in the Australian Breast Cancer Family Registry identified five carriers of *RNASEL:*p.Glu265* in 591 early onset breast cancer cases. Three of the five women (60%) carrying *RNASEL*:p.Glu265* also carried a pathogenic mutation in a breast cancer susceptibility gene compared with 30 carriers of pathogenic mutations in the 586 non-carriers of *RNASEL*:p.Glu265* (5%) (*p* < 0.002). Taqman genotyping demonstrated that the allele frequency of *RNASEL*:p.Glu265* was similar in affected and unaffected Australian women, consistent with other populations.

**Conclusion:**

Our study suggests that *RNASEL:*p.Glu265* may be a genetic modifier of risk for early-onset breast cancer predisposition in carriers of high-risk mutations. Much larger case-case and case-control studies are warranted to test the association observed in this report.

## Background

There is marked variability in individual cancer risk between and within *BRCA1* and *BRCA2* mutation carrier families [1, 2]. Accumulating evidence reviewed in [3] indicates that breast cancer risk in mutation carriers is modified by several risk factors that cluster in families, including genetic modifiers of risk that influence mutation penetrance. Segregation analyses studies have demonstrated that risk prediction models that allow for genes to modify effect on breast cancer risk in *BRCA1* and *BRCA2* mutation carriers fit significantly better to familial data than models without a modifying component.

**Fig. 1:**
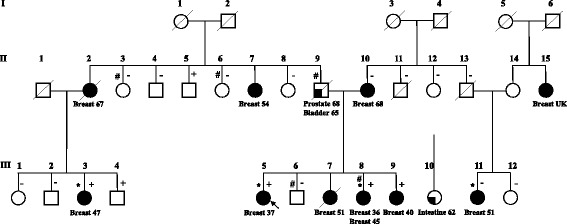
Pedigree of Family A (modified from Southey et al.*,* [5]). + and –: carriers and non-carriers of *PALB2*:p.Trp1038*, respectively (data from [1]); #: carriers of *RNASEL*:p.Glu265*; *: individuals selected for whole-exome sequencing; arrow: proband. Breast cancer is indicated by black filled symbols, and other cancers are indicated by quarter-filled symbols

Genetic modifiers of risk for carriers of high-risk mutations in other breast cancer susceptibility genes, such as *PALB2,* are yet to be described. In this study, we examined the exomes of key members of a multiple-case family segregating the pathogenic *PALB2*:c.3113G>A (p.Trp1038*) mutation (Family A, Fig. 1) to explore the possibility that additional genetic factors could be responsible for modifying the breast cancer risk in this family.

## Methods

### Subjects

The women in this study were ascertained via population-based sampling by the Australian site of the Breast Cancer Family Registry (ABCFR, [4]). Probands (defined as the first family members enrolled in the study, with or without a personal history of breast cancer) were identified from the Victoria and New South Wales cancer registries and invited to participate, regardless of family history.

All participants provided written informed consent for participation in this research program, which was approved by the ABCFR and the University of Melbourne Human Research Ethics Committee (Melbourne, VIC, Australia).

### Whole-exome sequencing

Whole-exome sequencing (WES) was performed on the germline DNA of four affected women from Family A: the proband (III.5) and her sister (III.8), one maternal cousin (III.3) and one paternal cousin (III.11) (Fig. 1). Three of these women were carriers of *PALB2*:c.3113G>A (p.Trp1038*). Details of the family, the breast cancer diagnoses and histology are described in Southey et al. [5]. WES and bioinformatics analysis were described by Park et al. [6]. Briefly, genetic variants were assessed for relevance to cancer etiology. The highest priority group for further investigation included nonsense and frameshift variants and genetic variants predicted to affect consensus splice sites. Variants in genes that have been associated with cancer predisposition, encode proteins involved in cell cycle checkpoint control or DNA repair pathways and confirmed by Sanger sequencing were prioritised. By applying this approach, we identified the truncating variant *RNASEL*:p.Glu265*, which was previously reported to increase prostate cancer risk, as a candidate modifier variant.

### Gene panel-testing

Gene-panel testing was performed on 591 probands participating to the ABCFR diagnosed under the age of 40 years who had biological sample available for testing.

Amplicon-based sequencing of the coding regions and proximal intron-exon junctions of *RNASEL* (NM_021133.3), *BRCA1* (NM_007294.3)*, BRCA2* (NM_000059.3) and *PALB2* (NM_024675.3) using the Hi-Plex protocol [7]. Massively parallel sequencing (150 bp paired-end) was performed on the MiSeq (Illumina, San Diego, CA, USA). Bioinformatics analysis and variant calling performed using ROVER as described in [8].

### Classification of variants

Classification of genetic variants in *BRCA1* and *BRCA2* was performed in accordance with the Evidence-based Network for the Interpretation of Germline Mutant Alleles (ENIGMA) consortium’s recommendations (April 2016 update) [9].

Consistent with ENIGMA classification criteria, all loss-of-function genetic variants in *PALB2* were considered “pathogenic”, unless there was evidence to the contrary.

Although *ATM* was not part of the panel-test, affected probands diagnosed under the age of 40 participating in the ABCFR have been genotyped for *ATM*:c.7271T>G by Chenevix-Trench et al. [10]. There is overwhelming data to support the association of this variant with breast cancer risk similar in magnitude to *BRCA2* mutations (e.g. [11]).

### Genotyping of RNASEL:P.Glu265*

*RNASEL:*p.E265* carrier frequency was determined by genotyping all probands participating in the ABCFR diagnosed with breast cancer regardless of age of onset (*n* = 1445, 591 of which were mutation-screened), and age-matched unaffected controls (*n* = 827). Clinical characteristics of the participants are presented in Table 1.

**Table 1:** Clinical characteristics of the participants to the population-based Australian Breast Cancer Family Registry that were genotyped for *RNASEL*:p.Glu265*

	Cases (*n* = 1445)	Controls (*n* = 827)
Age at diagnosis (years)	42 [23–59]	n/a
Age at recruitment (years)	43 [23–60]	41 [20–60]
Ethnicity		
White/Caucasian	1345 (93%)	710 (85.9%)
Asian and Pacific Islander	85 (5.9%)	32 (3.9%)
Other	8 (0.6%)	7 (0.8%)
Unknown	7 (0.5%)	78 (9.4%)
Laterality		
Right breast	713 (49.3%)	n/a
Left breast	732 (50.7%)	n/a
Estrogen Receptor Status		
Negative	439 (30.4%)	n/a
Positive	815 (56.4%)	n/a
Unknown	191 (13.2%)	n/a
Grade		
GI	212 (14.6%)	n/a
GII	526 (36.4%)	n/a
GIII	555 (38.4%)	n/a
GIV	3 (0.2%)	n/a
Unknown	149 (10.4%)	n/a

n/a: not applicable

GI, grade I; GII, grade II; GIII, Grade III; GIV, Grade IV

Genotyping was performed using a custom Taqman probe-based assay (ThermoFisher Scientific, Waltham, MA, USA) according to the manufacturer’s instructions. Probe sequences are available upon request. The reactions were amplified and analysed on the LightCycler480 (Roche, Penzberg, Germany).

### Statistical analysis

The difference in the prevalence of *RNASEL:*p.Glu265* in pathogenic mutation carriers and non-carriers (case only analysis) was tested using a two-sided Fisher’s Exact test.

## Results

### Whole-exome sequencing

The mutation *PALB2*:c.3113G>A (p.Trp1038*) was identified in the paternal lineage of Family A by Southey et al. [5]. There were ten diagnoses of breast cancer, five of which occurred under the age of 50 years, in nine women in the extended family (Fig. 1). WES was performed on four women and identified *RNASEL:*p.Glu265* in III.8, who is known to carry the *PALB2* mutation and had two primary diagnoses of breast cancer at age 36 and 45 years. Further genotyping identified *RNASEL:*p.Glu265* in four additional relatives unaffected by cancer. II.9 (an obligate carrier) had diagnoses of prostate and bladder cancer.

### Gene-panel testing

Panel-testing by targeted-sequencing of 591 early-onset affected probands from the ABCFR identified five additional carriers of *RNASEL:*p.Glu265*. No other pathogenic genetic variants (as per ENIGMA classification criteria) were observed in *RNASEL*.

Among the five carriers of *RNASEL:*p.Glu265*, there was one carrier of *BRCA2*:c.6275_6276delTT(p.Leu2092Profs), one carrier of *BRCA1:*c.4239del (p.Glu1413Aspfs) and one carrier of *ATM*:c.7271T>G (p.Val2424Glu) (previously identified by Chenevix-Trench [10]). In total, 3/5 early-onset affected probands carriers of *RNASEL:*p.Glu265* also harboured a pathogenic mutation in a known breast cancer susceptibility gene.

**Fig. 2:**
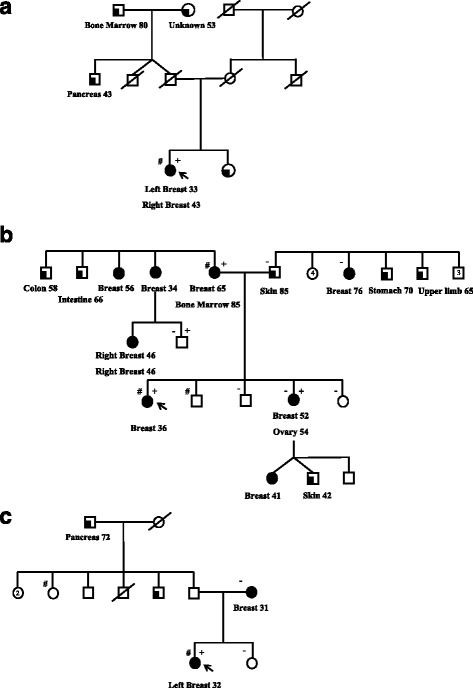
Pedigrees of the families carrying (**a**) *BRCA1*:p.Glu1413Aspfs, (**b**) *BRCA1*:p.Leu2092Profs and (**c**) *ATM*:p.Val2424Glu. +: carriers of the pathogenic mutation; # and -: carriers and non-carriers of *RNASEL*:p.Glu265*, respectively; arrow: proband. Breast cancer is indicated by black filled symbols, and other cancers are indicated by quarter-filled symbols. Numbers within symbols represent multiple individuals

Their family pedigrees are presented in Fig. 2. The family of the *BRCA1*:p.Glu1413Aspfs carrier could not be further tested (Fig. 2a). Mutation screening in the family carrying *BRCA2*:p.Glu1413Aspfs revealed two additional carriers of *RNASEL*:p.Glu265*: the proband’s mother, who had been diagnosed with breast cancer and leukemia (age at diagnoses 65 and 83 years respectively) and one of the proband’s brothers (Fig. 2b). The affected sister of the proband was found to carry *BRCA2*:p.Glu1413Aspfs but not the RNASEL mutation. In the family carrying *ATM*:p.Val2424Glu, *RNASEL*:p.Glu265* was inherited through the paternal side. One of the proband’s unaffected sisters who did not carry *ATM*:p.Val2424Glu was identified as a carrier of *RNASEL*:p.Glu265*.

Pathogenic mutations identified in the carriers and non-carriers of *RNASEL:*p.Glu265* are reported in Table 2. We identified 17 and 14 carriers of pathogenic mutations in *BRCA1* and *BRCA2*, respectively in affected probands in the ABCFR*.* One woman carried a mutation in both genes. No other loss-of-function *PALB2* mutation was identified in this early-onset breast cancer study.

**Table 2:** Pathogenic mutations^a^ identified by gene-panel testing in probands diagnosed before the age of 40 years in the population-based Australian Breast Cancer Family Registry

Gene ^b^	HGVS_c ^c^	HGVS_p^c^	Carriers
*BRCA1*	c.5266dupC	p.Gln1756Profs	4
	c.5095C > T	p.Arg1699Trp	1
	c.4689C > G	p.Tyr1563Ter	1
	c.4327C > T	p.Arg1443Ter	1
	c.4239del	p.Glu1413Aspfs	1
	c.4065_4068delTCAA	p.Asn1355Lysfs	2
	c.3756_3759delGTCT	p.Ser1253Argfs	1
	c.3155delA	p.Asn1052Metfs	1
	c.2681_2682delAA	p.Lys894Thrfs	2
	c.2475delC	p.Asp825Glufs	1
	c.1687C > T	p.Gln563Ter	1
	c.427G > T	p.Glu143Ter	1
	c.68_69delAG	p.Glu23Valfs	1^e^
*BRCA2*	c.250C > T	p.Gln84Ter	1
	c.755_758delACAG	p.Asp252Valfs	2
	c.3847_3848delGT	p.Val1283Lysfs	1
	c.5576_5579delTTAA	p.Ile1859Lysfs	1
	c.5946delT	p.Ser1982Argfs	3
	c.6275_6276delTT	p.Leu2092Profs	2
	c.8575delC	p.Gln2859Lysfs	3^e^
	c.8878C > T	p.Gln2960Ter	1
	c.8904delC	p.Val2969Cysfs	1
*ATM*	c.7271 T > G	p.Val2424Glu	1 ^d^

^a^Mutation in *BRCA1* and *BRCA2* that are classified as pathogenic by the expert panel ENIGMA, *PALB2:*p.Trp1038* or *ATM:*p.Val2424Glu

^b^Transcript sequences are *BRCA1*: NM_007294.3; *BRCA2*: NM_00059.3; *ATM*:NM_000051

^c^Variant nomenclature according to the Human Genome Variation Society (HGVS), HGVS_c for coding DNA and HGVS_p for protein variants

^d^Data from Chenevix-Trench et al.*,* [10]

^e^One woman carried these two mutations

Thus, in probands with early onset disease, the prevalence of RNASEL:p.Glu265* in carriers of a pathogenic mutation in a breast cancer susceptibility gene was 10% (3/30), compared to 0.36% (2/556) in non-carriers (*p* < 0.002, two-sided Fisher’s Exact test).

### Genotyping of RNASEL:P.Glu265*

Taqman-based genotyping identified 9/1445 (0.62%) breast cancer affected women and 6/817 (0.74%) unaffected controls who carry *RNASEL:*p.Glu265* indicating that the carrier frequency of *RNASEL:*p.Glu265* in Australian women was similar in affected probands and unaffected controls.

## Discussion

Whole-exome sequencing (WES) was performed on four women from Family A. On the basis of these results, we sought to investigate whether *RNASEL:*p.E265* could be a modifier of breast cancer susceptibility in high-risk mutation carriers.

*RNASEL* encodes the 2′,5′-oligoisoadenylate synthetase (2-5A)- dependent ribonuclease L (RNase L), an enzyme which has an antiviral role and may regulate the half-life of many mRNAs. The interferon viral response stimulates synthesis of 2-5A, which in turn stimulates activity of RNase L. The ribonuclease activity of RNase L inhibits proliferation of a variety of viruses. Additionally, continued activation of RNase L leads to degradation of 28S and 18S rRNA, which in turn activates a Jun-kinase-dependent apoptosis pathway [12–14]. An animal model of RNase L function showed that mice devoid of RNase L have defects in both interferon-induced apoptosis and antiviral response [12].

Carpten et al. identified *RNASEL* as a candidate prostate cancer susceptibility gene located within the Hereditary Prostate Cancer 1 (HPC1) linkage peak on chromosome 1q on the basis of evidence that two inactivating mutations in the gene, *RNASEL:*p.Met1Ile and *RNASEL:*p.Glu265*, segregated with prostate cancer in chromosome 1q–linked pedigrees [15]. In that study, the reported median age at prostate cancer onset was 11 years less in carriers of *RNASEL:*p.Glu265*. This variant is classified as pathogenic for prostate cancer susceptibility in ClinVar [16].

In a study of sporadic and familial pancreatic cancer, Bartsch et al. observed *RNASEL*:p.Glu265* in 1/36 (2.8%) pancreatic cancer cases with a family history of the disease, 1/75 (1.3%) pancreatic cancer cases without family history and none in 108 unaffected controls suggesting a possible association with pancreatic cancer susceptibility [17].

Some missense substitutions in *RNASEL* have been reported to interact with other genetic and environmental factors to increase early-onset risk of disease, e.g. *RNASEL:*c.1385G > A (p.Arg462Gln) and early-onset hereditary non-polyposis colorectal cancer in *MSH2* or *MLH1* pathogenic mutation carriers [18, 19]. Our study did not provide any evidence for a modifying role for *RNASEL*:p.Arg462Gln in breast cancer predisposition.

Genotyping of *RNASEL*:p.Glu265* in our case-control breast cancer study did not show an increased carrier frequency in breast cancer cases versus controls. However, our data indicate that in women with early-onset disease (diagnosed under 40 years old) the frequency of carriers of pathogenic mutations in a breast cancer susceptibility gene was significantly higher in *RNASEL*:p.Glu265* carriers than in *RNASEL*:p.Glu265* non-carriers.

The pathogenic mutations considered in this report are any *BRCA1* or *BRCA2* classified as pathogenic by ENIGMA, *PALB2*:p.Trp1038* and *ATM:*p.Val2424Glu. In this regard, it is notable that the only confirmed modifier of breast cancer risk, *RAD51:*c.135G > C, modifies risk only in *BRCA2* pathogenic mutation carriers [20]. Our findings suggest that *RNASEL:*p.Glu265* could be a genetic modifier of cancer predisposition for carriers of high-risk mutations in different breast cancer susceptibility genes*.*

Since cells from heterozygous carriers of *RNASEL:*p.Glu265* were shown to contain half the amount of RNase L [15], it is possible that this variant could induce a decreased apoptotic response. However, the mechanisms by which *RNASEL* could influence the risk of breast cancer are still unknown and should be further investigated.

Further work is required to test the hypothesis raised in this report. Studies of genetic modifiers utilising very large sample sizes can be achieved through the Consortium of Investigators of Modifiers of *BRCA1* and *BRCA2* (CIMBA) [3] who have collected DNA and epidemiological and clinical data for over 15,000 *BRCA1* carriers and 8,000 *BRCA2* carriers. Similar future studies related to *PALB2* mutation carriers could possibly be achieve within the *PALB2* Interest Group www.palb2.org.

## Conclusion

Here, we present new data that raises the possibility that *RNASEL:*p.Glu265* acts as a modifier of risk for carriers of rare high-risk genetic mutations. This case-only study report supports an interesting hypothesis that requires further testing in large case only and case-control studies.

Modifier genes/variants could partly explain inter-individual variation in risk between pathogenic mutation carriers. The identification of modifiers of breast cancer risk will help to refine individual risk estimates and optimise risk management.

## Abbreviations

ABCFR: Australian Breast Cancer Family Registry
*ATM*: ATM serine/threonine kinas
*BRCA1*: BRCA1, DNA repair associated
*BRCA2*: BRCA2, DNA repair associated
ENIGMA: Evidence-based Network for the Interpretation of Germline Mutant Alleles
HGVS: Human Genome Variation Society
*PALB2*: Partner and Localiser of BRCA2
*RNASEL*: Ribonuclease L
